# Opioid Free Ptyregopalatine Ganglion Block Based Multimodal Anesthesia Versus Conventional Opioid Based Multimodal Anesthesia for Tonsillectomy Operations: A Randomized Controlled Trial

**DOI:** 10.5812/aapm-146617

**Published:** 2024-07-10

**Authors:** Ramy Mahrose, Mohammed Sayed Shorbagy, Amr A. Kasem

**Affiliations:** 1 Assistant Professor of Anesthesiology, Intensive Care & Pain Management, Faculty of Medicine, Ain Shams University, Cairo, Egypt

**Keywords:** Opioid Free, Ptyregopalatine Ganglion, Multimodal Anesthesia, Opioid Based, Tonsillectomy

## Abstract

**Background:**

Otolaryngology often involves adenotonsillectomies, surgical procedures aimed at addressing obstructive sleep-disordered breathing and underlying apnea in children.

**Objectives:**

Pediatric anesthesiologists and otolaryngologists encounter challenges in managing pain post-adenotonsillectomy, especially due to the use of opioid medications in children who have an elevated baseline risk of airway obstruction and associated morbidity and mortality.

**Methods:**

This prospective, randomized, double-blinded study was conducted at Eldemerdash Hospital, Ain Shams University, Egypt, from September 2022 to August 2023. A total of 90 patients aged 5 - 13 years who underwent elective tonsillectomy surgery were randomly assigned to two groups, with 45 patients in each group. The first group, referred to as group A, received intravenous 1.0 μg/kg fentanyl, dexamethasone 150 μg/kg, and paracetamol 15 mg/kg. The second group, referred to as group B, received dexamethasone 150 μg/kg and paracetamol 15 mg/kg, with an opioid-free pterygopalatine ganglion block for multimodal anesthesia. The primary outcome measure of this study was the postoperative Wong-Baker scale at 1st, 3rd, and 24th hours.

**Results:**

This study included 90 individuals with similar demographic profiles and comparable initial characteristics and surgical techniques in both groups (P > 0.05). Intraoperative heart rate, mean blood pressure, additional analgesia, postoperative Wong-Baker scale, postoperative rescue analgesia, and laryngospasm did not show significant differences between the two groups (P > 0.05). However, there were statistically significant differences between the groups in terms of nausea, vomiting, hypoxia, and post-anesthesia care unit stay, with group B experiencing fewer of these issues (P < 0.05).

**Conclusions:**

Both conventional opioid-based multimodal anesthesia and opioid-free pterygopalatine ganglion block-based multimodal anesthesia are effective methods for providing analgesia during and after tonsillectomy surgery. The pterygopalatine ganglion block, the latter option, has been found to result in fewer postoperative complications such as nausea, vomiting, and hypoxia. Additionally, patients who receive this type of anesthesia typically require less time in the post-anesthesia care unit.

## 1. Background

Tonsillectomy is a highly prevalent surgical procedure among children in the United States, with over 530,000 performed annually. Many of these children experience sleep-disordered breathing or obstructive sleep apnea, making them particularly susceptible to respiratory depression. Moreover, the use of opioids can increase the likelihood of laryngospasm and airway swelling, which may intensify respiratory depressive effects (1).

Anesthesia and opioid exposure during childhood may also have neurotoxic effects on developing brains, causing neuronal apoptosis and long-term cognitive and behavioral deficits (2).

However, there has been a recent trend towards reducing the use of opioids in pediatric surgeries. In an effort to decrease perioperative opioid usage, several non-opioid medications have been utilized, such as dexamethasone, dexmedetomidine, lidocaine, and nitrous oxide (3).

Additionally, ibuprofen alone or in combination with acetaminophen can be used as painkillers to reduce the need for opioids. Careful utilization of these non-opioid options can improve healing rates and prevent the adverse effects of opioids without compromising patient comfort. However, the use of non-steroidal anti-inflammatory drugs such as ibuprofen and ketorolac in adenotonsillectomy is still debated due to concerns about post-operative bleeding (4).

Intranasal administration of xylocaine has the potential to block the pterygopalatine ganglion, thereby blocking palatopharyngeal sensations. This method can serve as a basis for opioid-free multimodal anesthesia in adenotonsillectomy procedures (5).

This method likely blocks the pterygopalatine ganglion and the maxillary division of the trigeminal nerve to some extent. Direct application of medicine to numb the tonsil bed from behind the nose can help control pain and nausea after tonsillectomy, even though systemic absorption of nasal lignocaine remains a consideration (6).

## 2. Objectives

In this study, we hypothesize that opioid-free anesthesia based on pterygopalatine ganglion block is superior to conventional opioid-based multimodal anesthesia for tonsillectomy operations. Our aim is to assess its impact on intraoperative hemodynamics, postoperative opioid administration, pain scores, rates of nausea and vomiting, hypoxia, and length of stay in the post anesthesia care unit (PACU).

## 3. Methods

This prospective, randomized (using simple randomization), double-blinded (participants and study staff, including data collectors), controlled study was conducted at Eldemerdash Hospital. The study was carried out from September 2022 to August 2023 following approval from the Research Ethical Committee of Ain Shams University (No. IRB 00006379) and clinical trial registration (NCT05513209). Informed consent was obtained from the guardians of the patients prior to the procedure. Using a sealed envelope randomized technique, a total of 90 patients aged 5 to 13 years undergoing elective tonsillectomy surgery were included in the study. Exclusion criteria included allergies to xylocaine, developmental delays, severe cognitive impairment, American Society of Anesthesiologists (ASA) class 3 and 4, suspected difficult intubation, and concurrent procedures like lingual tonsillectomy.

Pre-operatively, all patients underwent a thorough assessment, including a detailed medical history, comprehensive physical examination, and various laboratory tests such as complete blood count (CBC), prothrombin time (PT), partial thromboplastin time (PTT), prothrombin concentration (PC), and erythrocyte sedimentation rate (ESR).

During the intraoperative period, all patients received standard monitoring, including noninvasive blood pressure (NIBP), peripheral oxygen saturation (SPO_2_), and electrocardiography (ECG).

A computerized program was used to randomly assign all patients to one of two equal groups, with each group consisting of forty-five patients.

### 3.1. Group A (Opioid Based Multimodal Anesthesia)

General anesthesia was induced by inhalational administration of sevoflurane (2 - 5%) in a mixture of oxygen and air, with a gradual increase in sevoflurane concentration during induction. Following induction, a 22-gauge cannula was inserted to administer intravenous atropine at a dose of 0.01 mg/kg, paracetamol at a dose of 15 mg/kg, dexamethasone at a dose of 150 μg/kg, fentanyl at a dose of 1.0 μg/kg, and suxamethonium at a dose of 1.0 mg/kg as a muscle relaxant for endotracheal intubation. Anesthesia was maintained with sevoflurane in a 50% mixture of O_2_/air and intermittent positive pressure ventilation to keep the end-tidal carbon dioxide around 35 mmHg.

### 3.2. Group B (Opioid Free Ptyregopalatine Ganglion Block Based Multimodal Anesthesia)

General anesthesia was performed as in group A, but without the use of fentanyl. Next, the sphenopalatine ganglion block (SPGB) was executed in an upright position with an extended neck and upward nasal projections. A total of 2 mL of 2% lignocaine (with a maximum dose not exceeding 4.5 mg/kg) was gradually instilled from the topmost part of the middle concha to each nostril's posterior wall. We gently pressed the nostrils to prevent the drug from seeping out. Blocking takes about 5 minutes. After this period, the operation was started (7).

After the operation was completed, patients regained spontaneous breathing once the inhalational anesthetic was stopped. They were then extubated after regaining consciousness and having their oropharyngeal secretions suctioned.

**Figure 1.**
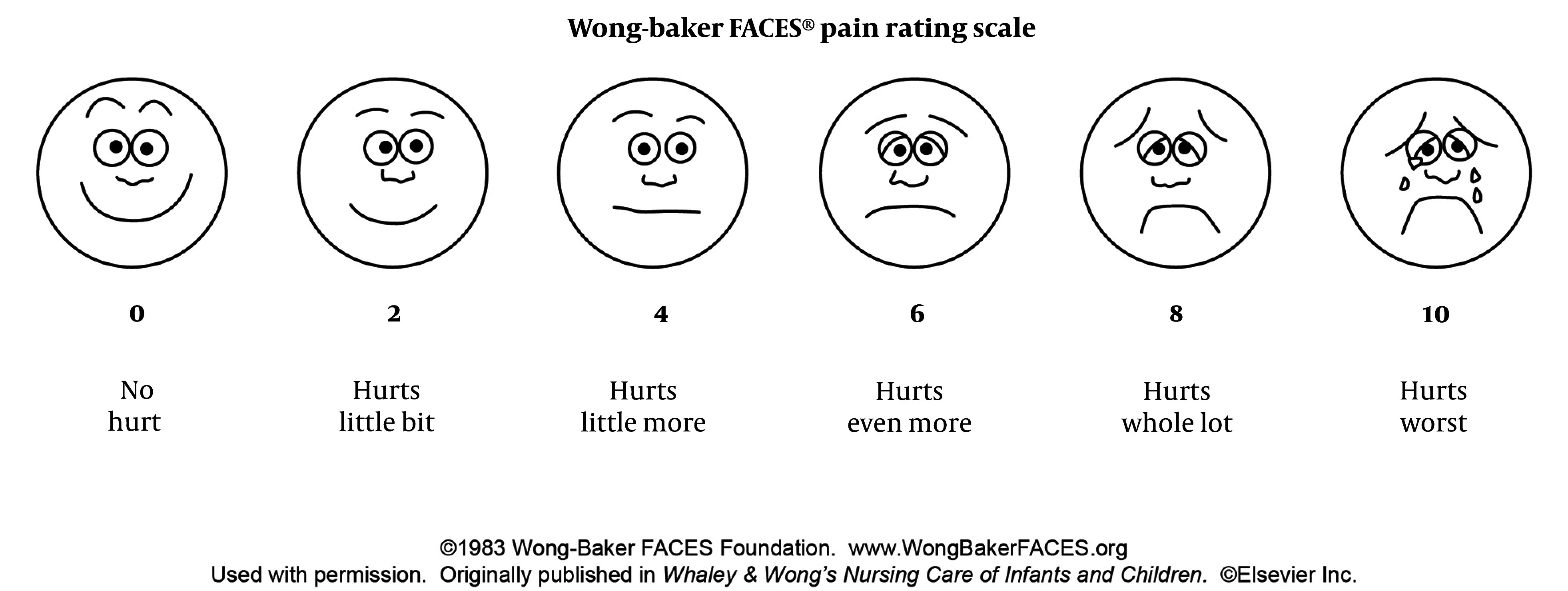
Wong Baker scale

The following data was recorded for each patient: Intraoperative heart rate and blood pressure were recorded every 5 minutes until the end of the operation; any increase in heart rate or blood pressure exceeding 20% from the baseline; and the administration of additional intraoperative analgesia in the form of fentanyl (1 μg per kg). Postoperatively, the Wong-Baker scale (Figure 1) was used to assess pain levels at the 1st, 3rd, and 24th hours. If the score was 4 or higher, postoperative analgesia in the form of tramadol (1 mg per kg) was administered. Other recorded variables included postoperative (during 24 hours) nausea, vomiting, hypoxia, and the length of stay in the PACU.

The Wong-Baker scale, which consists of a series of faces ranging from a happy face at 0, representing "no pain," to a crying face at 10, representing "the worst pain imaginable," was utilized by the physician. The scale was then explained to the patient's guardian, typically the mother, for use over the duration of 24 hours following the patient's discharge from the hospital.

### 3.3. Outcome Measures

#### 3.3.1. 1ry Outcome

Postoperative pain will be measured using the Wong-Baker scale at the 1st, 3rd, and 24th hours.

#### 3.3.2. 2ry Outcome

During the intra-operative period, it is important to consider the need for additional analgesia and to monitor mean systemic blood pressure and pulse rate. Postoperatively, it is crucial to provide rescue analgesia and be aware of potential complications such as nausea, vomiting, hypoxia, laryngeal spasm, and the duration of stay in the PACU.

### 3.4. Sample Size Calculation

The analysis was done using the PASS 11 program. A significance level (alpha) of 0.05 was achieved by obtaining an 80% power with group sample sizes of 45 each, assuming the real distribution is equal, using a two-sided Mann-Whitney test.

### 3.5. Statistical Analysis

The statistical package for social sciences, IBM SPSS version 23, was utilized to analyze the data. Means and standard deviations or ranges were used to represent quantitative variables, while medians, including the inter-quartile range (IQR), were used for non-parametric mean values. Numbers and percentages were employed to represent qualitative variables. Quantitative data with a non-parametric distribution were compared between the two study groups using the Mann-Whitney test. Qualitative data were compared between different groups using the chi-square test. The confidence interval was 95%, and the margin of error was 5%. A P-value less than 0.05 was considered significant, less than 0.01 was considered highly significant, and greater than 0.05 was considered not significant.

## 4. Results

**Figure 2.**
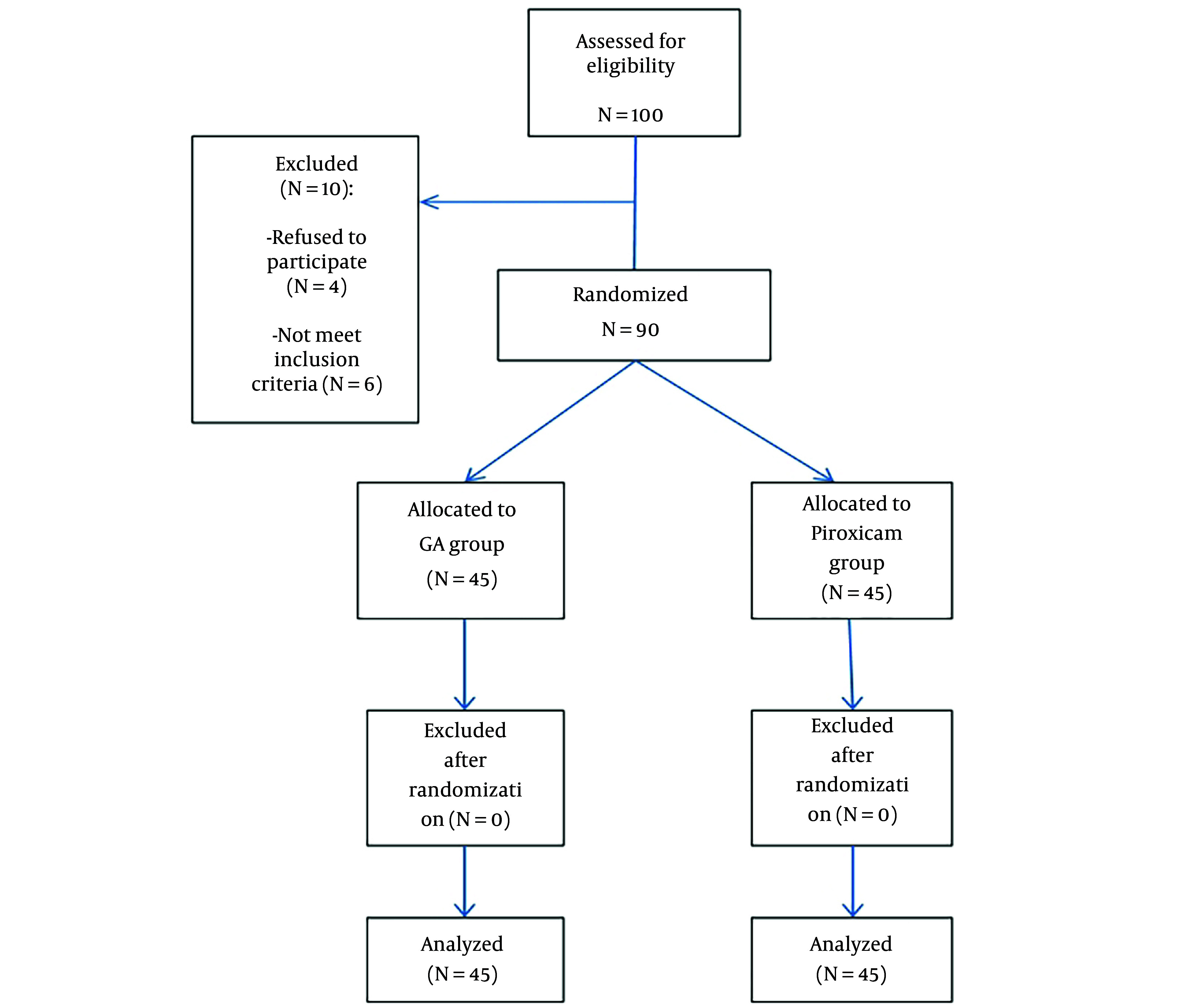
Study CONSORT flow chart.

The CONSORT flow diagram of the study, in which 90 patients were enrolled and analyzed, is shown in Figure 2. 

### 4.1. Demographic Data

There were no significant statistical differences in the demographic data of either group (Table 1). 

**Table 1.** Patients Demographic Data ^a, b^

Variables	Group A	Group B	P-Value ^c^
**Age (y)**	8.4 ± 2.1	8.1 ± 2.4	0.465
**Sex (M/F)**	22/23	23/22	0.77/0.77
**Weight**	27.9 ± 4.4	27.4 ± 4.2	0.563
**Duration of the operation (minutes)**	31.4 ± 5	32.2 ± 4.7	0.465

^a^ Values are expressed as mean ± standard deviation or numbers.

^b^ Using student’s *t*-test and chi-square test.

^c^ P-value > 0.05: Non-significant (NS).

### 4.2. Intraoperative Heart Rate, Mean Arterial Blood Pressure and Additional Analgesia

The intraoperative heart rate, mean arterial blood pressure, and additional analgesia in either group did not show any significant statistical differences (Table 2). 

**Table 2.** Intraoperative Mean Heart Rate, Mean Arterial Blood Pressure and Additional Analgesia ^a, b^

Variables	Group A	Group B	P-Value ^c^
**Mean heart rate (beat per minute)**	88.8 ± 7.6	86.3 ± 7.8	0.129
**Mean arterial blood pressure (mm mercury)**	65 ± 3.1	65 ± 3.3	0.922
**Intraoperative additional analgesia**	2 (4.4)	3 (6.7)	0.702

^a^ Values are expressed as mean ± standard deviation or No. (%).

^b^ Using student’s *t*-test and chi-square test.

^c^ P-value > 0.05: Non-significant (NS).

### 4.3. Postoperative Wong Baker Scale and Additional Analgesia

The two groups did not exhibit any significant statistical differences concerning postoperative Wong-Baker scale scores at the first, third, and 24th hours postoperatively. In group A, 5 patients experienced significant postoperative pain with a Wong-Baker score of 4 or higher, and they were given additional postoperative analgesia in the form of tramadol 1 mg/kg. In group B, 4 patients also suffered from significant postoperative pain with a Wong-Baker score of 4 or higher, and they were also given additional postoperative analgesia in the form of tramadol 1 mg/kg. The difference in pain and analgesia between the two groups was not statistically significant, as shown in Table 3. 

**Table 3.** Postoperative Wong Baker Scale and Additional Analgesia ^a, b^

Variables	Group A	Group B	P-Value ^c^
**Postoperative Wong Baker scale**			
1st hour	2 (0 - 2)	2 (0 - 2)	0.515
3rd hour	2 (0 - 2)	2 (0 - 2)	0.262
24th hour	2 (0 - 2)	2 (1.5 - 2)	0.464
**Postoperative additional analgesia**	5 (11.1)	4 (8.9)	0.725

^a^ Values are expressed as median (interquartile range) or No. (%).

^b^ Using Mann-Whitney U test and chi-square test.

^c^ P-value > 0.05: Non-significant (NS).

### 4.4. Postoperative Complications (Nausea, Vomiting, Hypoxia, Laryngeal Spasm) and the Length of PACU Stay

There were significant statistical differences between the two groups regarding postoperative nausea, vomiting, hypoxia, and the length of PACU stay. Specifically, these factors were higher in group A compared to group B. However, there were no significant statistical differences between the two groups in relation to postoperative laryngeal spasm, as shown in Table 4. 

**Table 4.** Postoperative Complications (Nausea, Vomiting, Hypoxia, Laryngeal Spasm and the Length of Post Anesthesia Care Unit Stay) ^a, b^

Postoperative Complications	Group A	Group B	P-Value ^c^
**Nausea**	10 (22.2)	3 (6.6)	0.036
**Vomiting**	8 (17.7)	2 (4.4)	0.045
**Hypoxia**	7 (15.5)	1 (2.2)	0.027
**Laryngeal spasm**	2 (4.4)	1 (2.2)	0.561
**PACU length of stay (minutes) **	146.7 ± 9.3	80.4 ± 8.4	< 0.0001

^a^ Values are expressed as Mean ± standard deviation or No. (%).

^b^ Using student’s *t*-test and chi-square test.

^c^ P-value > 0.05: Non-significant (NS); P-value < 0.05: Significant (S); P-value < 0.01: Highly significant (HS).

## 5. Discussion

In pediatric tonsillectomies, acute pain is a difficult issue due to the increased prevalence of sleep-disordered breathing and obstructive sleep apnea. Anesthesiologists may consciously choose not to prescribe opioids, especially in patients with severe obstructive sleep apnea. To ensure accurate results, the study excluded patients classified as ASA 3 - 4, thereby eliminating those with severe obstructive sleep apnea (8).

This study discovered that 41 patients (91.1%) who received an opioid-free intraoperative regimen also had no need for opioids following surgery. This outcome indicates that the surgical procedure could be achieved without the need for opioids. Postoperative vomiting is a significant concern for both parents and patients, as it can lead to complications and may require readmission (9). Our study revealed that 22.2% of patients in group A experienced nausea, compared to 6.6% of patients in group B, with a significant statistical difference. Additionally, the study found that 17.7% of patients in group A and 4.4% of patients in group B developed vomiting after tonsillectomy, with a significant statistical difference between the two groups.

Mann et al. (8) observed that a comparable percentage of patients in both groups (28.4% opioid vs. 33.7% opioid-free) did not need any analgesics after the operation. The results indicate that pain control levels were similar regardless of the use of intraoperative opioids. These findings align with our own results, which revealed no significant difference between the two groups in terms of the need for rescue analgesia after tonsillectomy. This suggests that a pterygopalatine ganglion block is as effective as opioids in managing postoperative pain.

Franz et al. (10) conducted a quality improvement project to explore effective opioid-free analgesic strategies for tonsillectomy. The findings revealed that 91.1% of patients managed to remain opioid-free throughout the entire procedure, both intraoperatively and postoperatively. These results strongly support the adoption of an initial analgesic approach for tonsillectomy that completely avoids opioids and instead incorporates dexamethasone and acetaminophen as adjuncts.

Cho et al. (11) reported that the SPGB has limited usefulness for postoperative pain relief in patients undergoing functional endoscopic sinus surgery. These findings contradict our own results, which demonstrate the effectiveness of the sphenopalatine ganglion block for postoperative analgesia following tonsillectomy. The disparity in outcomes may be due to the differing nature of the surgical procedures.

Kesimci et al. (12) reported that the use of either bupivacaine or levobupivacaine for sphenopalatine ganglion block effectively provides postoperative analgesia after functional endoscopic sinus surgery. This finding aligns with our own results.

Lin et al. (13) studied bilateral suprazygomatic infratemporal pterygopalatine fossa injections, previously known as suprazygomatic maxillary nerve blocks. These injections are a common treatment for children who may be at risk of respiratory complications and are more sensitive to opioids. The study revealed successful pain control and considered these injections a safer alternative to opioids. Our findings support these outcomes, although we specifically focused on the pterygopalatine ganglion block using nasal instillation of a local anesthetic.

Bhargava et al. (14) found that it is easier and faster to use a syringe or dropper to administer local anesthetic, which effectively blocks the sphenopalatine ganglion. Our study relied on this approach to successfully perform pterygopalatine ganglion blocks, yielding satisfactory results. Additionally, the systemic absorption of nasal lignocaine can help control pain and nausea after tonsillectomy.

Mulier (15) suggested that opioid-free anesthesia is a beneficial option for reducing nausea and vomiting after surgery, without any negative impact on pain levels or patient safety. This could be attributed to the decrease in opioid usage, which is often associated with postoperative nausea and vomiting. Our study results support this notion, as we observed a lower incidence of nausea, vomiting, and hypoxia in the group that received opioid-free anesthesia with pterygopalatine ganglion block-based multimodal anesthesia.

The length of stay in the PACU was found to be longer for the group that received conventional opioid-based anesthesia. This suggests that patients who did not receive opioids during surgery may have experienced fewer adverse events compared to those who did receive opioids (16). Additionally, a similar percentage of patients in both groups (88.9% in the opioid group and 91.1% in the opioid-free group) did not need any postoperative pain medication. This further supports the notion that pain control levels were comparable, regardless of whether opioids were administered during surgery.

### 5.1. Limitations

While our study evaluates immediate postoperative outcomes, it is crucial to conduct longitudinal assessments of neurodevelopmental trajectories, cognitive function, and behavioral outcomes beyond the immediate postoperative period to identify potential neurocognitive sequelae associated with different anesthesia techniques. Long-term follow-up would provide valuable information on the sustained neurological effects and safety profile of our study interventions. Further studies using the intranasal xylocaine instillation method for children undergoing tonsillectomy are needed, with larger sample sizes to prove the effectiveness and suitability of the block in children.

### 5.2. Conclusions

Both conventional opioid-based multimodal anesthesia and opioid-free pterygopalatine ganglion block-based multimodal anesthesia are effective for providing analgesia during and after surgery. However, postoperative complications such as nausea, vomiting, and hypoxia are less common with the pterygopalatine ganglion block-based approach, resulting in a shorter stay in the post-anesthesia care unit.

## Data Availability

The data sets used during the current study are available from the corresponding author on reasonable request.

## Clinical Trial Registration Code:

NCT05513209.
